# Supplementary material for: Two-Period Study Results from a Large Italian Hospital Laboratory Attesting SARS-CoV-2 Variant PCR Assay Evolution
Source: Microbiol Spectr. 2022 Nov 21;10(6):e02922-22. doi: 10.1128/spectrum.02922-22 (PMC9769628; doi:10.1128/spectrum.02922-22)
Supplement: Supplemental file 1 — Tables S1 and S2. Download spectrum.02922-22-s0001.pdf, PDF file, 0.2 MB [file spectrum.02922-22-s0001.pdf]

Details about the SARS-CoV-2 variant identification in 365 nasopharyngeal swab samples

Sample Name	Sequencing Method	Sequening Kit/Assay	Sequence Analysis Software/Platform	WHO/CDC Designation	Pango Lineage Designation
LAZ-FPG-044	WGS	Paragon Genomics CleanPlex SARS-CoV-2 panel	SOPHiA GENETICS platform for COVID-19	Delta	B.1.617.2
LAZ-FPG-045	WGS	Paragon Genomics CleanPlex SARS-CoV-2 panel	SOPHiA GENETICS platform for COVID-20	Delta	B.1.617.2
LAZ-FPG-046	WGS	Paragon Genomics CleanPlex SARS-CoV-2 panel	SOPHiA GENETICS platform for COVID-21	Delta	B.1.617.2
LAZ-FPG-047	WGS	Paragon Genomics CleanPlex SARS-CoV-2 panel	SOPHiA GENETICS platform for COVID-22	Delta	B.1.617.2
LAZ-FPG-048	WGS	Paragon Genomics CleanPlex SARS-CoV-2 panel	SOPHiA GENETICS platform for COVID-23	Gamma	P1
LAZ-FPG-049	WGS	Paragon Genomics CleanPlex SARS-CoV-2 panel	SOPHiA GENETICS platform for COVID-24	Delta	B.1.617.2
LAZ-FPG-050	WGS	Paragon Genomics CleanPlex SARS-CoV-2 panel	SOPHiA GENETICS platform for COVID-25	Delta	B.1.617.2
LAZ-FPG-051	WGS	Paragon Genomics CleanPlex SARS-CoV-2 panel	SOPHiA GENETICS platform for COVID-26	Delta	B.1.617.2
LAZ-FPG-052	WGS	Paragon Genomics CleanPlex SARS-CoV-2 panel	SOPHiA GENETICS platform for COVID-27	Delta	B.1.617.2
LAZ-FPG-053	WGS	Paragon Genomics CleanPlex SARS-CoV-2 panel	SOPHiA GENETICS platform for COVID-28	Delta	B.1.617.2
LAZ-FPG-054	WGS	Paragon Genomics CleanPlex SARS-CoV-2 panel	SOPHiA GENETICS platform for COVID-29	Delta	B.1.617.2
LAZ-FPG-055	WGS	Paragon Genomics CleanPlex SARS-CoV-2 panel	SOPHiA GENETICS platform for COVID-30	Delta	B.1.617.2
LAZ-FPG-056	WGS	Paragon Genomics CleanPlex SARS-CoV-2 panel	SOPHiA GENETICS platform for COVID-31	Delta	B.1.617.2
LAZ-FPG-058	WGS	Paragon Genomics CleanPlex SARS-CoV-2 panel	SOPHiA GENETICS platform for COVID-32	Delta	B.1.617.2
LAZ-FPG-059	WGS	Paragon Genomics CleanPlex SARS-CoV-2 panel	SOPHiA GENETICS platform for COVID-33	Delta	B.1.617.2
LAZ-FPG-060	WGS	Paragon Genomics CleanPlex SARS-CoV-2 panel	SOPHiA GENETICS platform for COVID-34	Delta	B.1.617.2
LAZ-FPG-061	WGS	Paragon Genomics CleanPlex SARS-CoV-2 panel	SOPHiA GENETICS platform for COVID-35	Gamma	P1
LAZ-FPG-062	WGS	Paragon Genomics CleanPlex SARS-CoV-2 panel	SOPHiA GENETICS platform for COVID-36	Delta	B.1.617.2
LAZ-FPG-063	WGS	Paragon Genomics CleanPlex SARS-CoV-2 panel	SOPHiA GENETICS platform for COVID-37	Delta	B.1.617.2
LAZ-FPG-064	WGS	Paragon Genomics CleanPlex SARS-CoV-2 panel	SOPHiA GENETICS platform for COVID-38	Delta	B.1.617.2
LAZ-FPG-065	WGS	Paragon Genomics CleanPlex SARS-CoV-2 panel	SOPHiA GENETICS platform for COVID-39	Delta	B.1.617.2
LAZ-FPG-066	WGS	Paragon Genomics CleanPlex SARS-CoV-2 panel	SOPHiA GENETICS platform for COVID-40	Delta	B.1.617.2
LAZ-FPG-067	WGS	Paragon Genomics CleanPlex SARS-CoV-2 panel	SOPHiA GENETICS platform for COVID-41	Delta	B.1.617.2
LAZ-FPG-068	WGS	Paragon Genomics CleanPlex SARS-CoV-2 panel	SOPHiA GENETICS platform for COVID-42	Delta	B.1.617.2
LAZ-FPG-082	WGS	Paragon Genomics CleanPlex SARS-CoV-2 panel	SOPHiA GENETICS platform for COVID-43	Delta	B.1.617.2
LAZ-FPG-083	WGS	Paragon Genomics CleanPlex SARS-CoV-2 panel	SOPHiA GENETICS platform for COVID-44	Delta	B.1.617.2
LAZ-FPG-084	WGS	Paragon Genomics CleanPlex SARS-CoV-2 panel	SOPHiA GENETICS platform for COVID-45	Delta	B.1.617.2
LAZ-FPG-085	WGS	Paragon Genomics CleanPlex SARS-CoV-2 panel	SOPHiA GENETICS platform for COVID-46	Delta	B.1.617.2
LAZ-FPG-086	WGS	Paragon Genomics CleanPlex SARS-CoV-2 panel	SOPHiA GENETICS platform for COVID-47	Delta	B.1.617.2
LAZ-FPG-087	WGS	Paragon Genomics CleanPlex SARS-CoV-2 panel	SOPHiA GENETICS platform for COVID-48	Delta	B.1.617.2
LAZ-FPG-088	WGS	Paragon Genomics CleanPlex SARS-CoV-2 panel	SOPHiA GENETICS platform for COVID-49	Delta	B.1.617.2
LAZ-FPG-090	WGS	Paragon Genomics CleanPlex SARS-CoV-2 panel	SOPHiA GENETICS platform for COVID-50	Delta	B.1.617.2
LAZ-FPG-091	WGS	Paragon Genomics CleanPlex SARS-CoV-2 panel	SOPHiA GENETICS platform for COVID-51	Delta	B.1.617.2
LAZ-FPG-123	WGS	Paragon Genomics CleanPlex SARS-CoV-2 panel	SOPHiA GENETICS platform for COVID-52	Delta	B.1.617.2
LAZ-FPG-124	WGS	Paragon Genomics CleanPlex SARS-CoV-2 panel	SOPHiA GENETICS platform for COVID-53	Delta	B.1.617.2
LAZ-FPG-125	WGS	Paragon Genomics CleanPlex SARS-CoV-2 panel	SOPHiA GENETICS platform for COVID-54	Delta	B.1.617.2
LAZ-FPG-126	WGS	Paragon Genomics CleanPlex SARS-CoV-2 panel	SOPHiA GENETICS platform for COVID-55	Delta	B.1.617.2
LAZ-FPG-127	WGS	Paragon Genomics CleanPlex SARS-CoV-2 panel	SOPHiA GENETICS platform for COVID-56	Delta	B.1.617.2
LAZ-FPG-128	WGS	Paragon Genomics CleanPlex SARS-CoV-2 panel	SOPHiA GENETICS platform for COVID-57	Delta	B.1.617.2
LAZ-FPG-129	WGS	Paragon Genomics CleanPlex SARS-CoV-2 panel	SOPHiA GENETICS platform for COVID-58	Delta	B.1.617.2
LAZ-FPG-130	WGS	Paragon Genomics CleanPlex SARS-CoV-2 panel	SOPHiA GENETICS platform for COVID-59	Delta	B.1.617.2
LAZ-FPG-131	WGS	Paragon Genomics CleanPlex SARS-CoV-2 panel	SOPHiA GENETICS platform for COVID-60	Delta	B.1.617.2
LAZ-FPG-132	WGS	Paragon Genomics CleanPlex SARS-CoV-2 panel	SOPHiA GENETICS platform for COVID-61	Delta	B.1.617.2

LAZ-FPG-133	WGS	Paragon Genomics CleanPlex SARS-CoV-2 panel	SOPHiA GENETICS platform for COVID-62	Delta	B.1.617.2
LAZ-FPG-134	WGS	Paragon Genomics CleanPlex SARS-CoV-2 panel	SOPHiA GENETICS platform for COVID-63	Delta	B.1.617.2
LAZ-FPG-135	WGS	Paragon Genomics CleanPlex SARS-CoV-2 panel	SOPHiA GENETICS platform for COVID-64	Delta	B.1.617.2
LAZ-FPG-136	WGS	Paragon Genomics CleanPlex SARS-CoV-2 panel	SOPHiA GENETICS platform for COVID-65	Delta	B.1.617.2
LAZ-FPG-137	WGS	Paragon Genomics CleanPlex SARS-CoV-2 panel	SOPHiA GENETICS platform for COVID-66	Delta	B.1.617.2
LAZ-FPG-138	WGS	Paragon Genomics CleanPlex SARS-CoV-2 panel	SOPHiA GENETICS platform for COVID-67	Delta	B.1.617.2
LAZ-FPG-139	WGS	Paragon Genomics CleanPlex SARS-CoV-2 panel	SOPHiA GENETICS platform for COVID-68	Delta	B.1.617.2
LAZ-FPG-140	WGS	Paragon Genomics CleanPlex SARS-CoV-2 panel	SOPHiA GENETICS platform for COVID-69	Delta	B.1.617.2
LAZ-FPG-141	WGS	Paragon Genomics CleanPlex SARS-CoV-2 panel	SOPHiA GENETICS platform for COVID-70	Delta	B.1.617.2
LAZ-FPG-146	WGS	Paragon Genomics CleanPlex SARS-CoV-2 panel	SOPHiA GENETICS platform for COVID-71	Alpha	B.1.1.7
LAZ-FPG-147	WGS	Paragon Genomics CleanPlex SARS-CoV-2 panel	SOPHiA GENETICS platform for COVID-72	Alpha	B.1.1.7
LAZ-FPG-148	WGS	Paragon Genomics CleanPlex SARS-CoV-2 panel	SOPHiA GENETICS platform for COVID-73	Alpha	B.1.1.7
LAZ-FPG-149	WGS	Paragon Genomics CleanPlex SARS-CoV-2 panel	SOPHiA GENETICS platform for COVID-74	Alpha	B.1.1.7
LAZ-FPG-150	WGS	Paragon Genomics CleanPlex SARS-CoV-2 panel	SOPHiA GENETICS platform for COVID-75	Alpha	B.1.1.7
LAZ-FPG-152	WGS	Paragon Genomics CleanPlex SARS-CoV-2 panel	SOPHiA GENETICS platform for COVID-76	Alpha	B.1.1.7
LAZ-FPG-153	WGS	Paragon Genomics CleanPlex SARS-CoV-2 panel	SOPHiA GENETICS platform for COVID-77	Alpha	B.1.1.7
LAZ-FPG-154	WGS	Paragon Genomics CleanPlex SARS-CoV-2 panel	SOPHiA GENETICS platform for COVID-78	Alpha	B.1.1.7
LAZ-FPG-156	WGS	Paragon Genomics CleanPlex SARS-CoV-2 panel	SOPHiA GENETICS platform for COVID-79	Alpha	B.1.1.7
LAZ-FPG-157	WGS	Paragon Genomics CleanPlex SARS-CoV-2 panel	SOPHiA GENETICS platform for COVID-80	Alpha	B.1.1.7
LAZ-FPG-158	WGS	Paragon Genomics CleanPlex SARS-CoV-2 panel	SOPHiA GENETICS platform for COVID-81	Alpha	B.1.1.7
LAZ-FPG-160	WGS	Paragon Genomics CleanPlex SARS-CoV-2 panel	SOPHiA GENETICS platform for COVID-82	Delta	B.1.617.2
LAZ-FPG-161	WGS	Paragon Genomics CleanPlex SARS-CoV-2 panel	SOPHiA GENETICS platform for COVID-83	Delta	B.1.617.2
LAZ-FPG-162	WGS	Paragon Genomics CleanPlex SARS-CoV-2 panel	SOPHiA GENETICS platform for COVID-84	Alpha	B.1.1.7
LAZ-FPG-163	WGS	Paragon Genomics CleanPlex SARS-CoV-2 panel	SOPHiA GENETICS platform for COVID-85	Alpha	B.1.1.7
LAZ-FPG-164	WGS	Illumina COVIDSeq assay	Illumina DRAGEN COVIDSeq Pipeline	Delta	B.1.617.2
LAZ-FPG-165	WGS	Illumina COVIDSeq assay	Illumina DRAGEN COVIDSeq Pipeline	Delta	B.1.617.2
LAZ-FPG-166	WGS	Illumina COVIDSeq assay	Illumina DRAGEN COVIDSeq Pipeline	Delta	B.1.617.2
LAZ-FPG-167	WGS	Illumina COVIDSeq assay	Illumina DRAGEN COVIDSeq Pipeline	Delta	B.1.617.2
LAZ-FPG-168	WGS	Illumina COVIDSeq assay	Illumina DRAGEN COVIDSeq Pipeline	Delta	B.1.617.2
LAZ-FPG-209	WGS	Illumina COVIDSeq assay	Illumina DRAGEN COVIDSeq Pipeline	Delta	B.1.617.2
LAZ-FPG-210	WGS	Illumina COVIDSeq assay	Illumina DRAGEN COVIDSeq Pipeline	Delta	B.1.617.2
LAZ-FPG-211	WGS	Illumina COVIDSeq assay	Illumina DRAGEN COVIDSeq Pipeline	Delta	B.1.617.2
LAZ-FPG-212	WGS	Illumina COVIDSeq assay	Illumina DRAGEN COVIDSeq Pipeline	Delta	B.1.617.2
LAZ-FPG-213	WGS	Illumina COVIDSeq assay	Illumina DRAGEN COVIDSeq Pipeline	Delta	B.1.617.2
LAZ-FPG-214	WGS	Illumina COVIDSeq assay	Illumina DRAGEN COVIDSeq Pipeline	Delta	B.1.617.2
LAZ-FPG-215	WGS	Illumina COVIDSeq assay	Illumina DRAGEN COVIDSeq Pipeline	Delta	B.1.617.2
LAZ-FPG-216	WGS	Illumina COVIDSeq assay	Illumina DRAGEN COVIDSeq Pipeline	Delta	B.1.617.2
LAZ-FPG-217	WGS	Illumina COVIDSeq assay	Illumina DRAGEN COVIDSeq Pipeline	Delta	B.1.617.2
LAZ-FPG-218	WGS	Illumina COVIDSeq assay	Illumina DRAGEN COVIDSeq Pipeline	Delta	B.1.617.2
LAZ-FPG-219	WGS	Illumina COVIDSeq assay	Illumina DRAGEN COVIDSeq Pipeline	Delta	B.1.617.2
LAZ-FPG-220	WGS	Illumina COVIDSeq assay	Illumina DRAGEN COVIDSeq Pipeline	Delta	B.1.617.2
LAZ-FPG-221	WGS	Illumina COVIDSeq assay	Illumina DRAGEN COVIDSeq Pipeline	Delta	B.1.617.2
LAZ-FPG-222	WGS	Illumina COVIDSeq assay	Illumina DRAGEN COVIDSeq Pipeline	Delta	B.1.617.2
LAZ-FPG-223	WGS	Illumina COVIDSeq assay	Illumina DRAGEN COVIDSeq Pipeline	Delta	B.1.617.2
LAZ-FPG-254	WGS	Illumina COVIDSeq assay	Illumina DRAGEN COVIDSeq Pipeline	Delta	B.1.617.2

LAZ-FPG-255	WGS	Illumina COVIDSeq assay	Illumina DRAGEN COVIDSeq Pipeline	Delta	B.1.617.2
LAZ-FPG-256	WGS	Illumina COVIDSeq assay	Illumina DRAGEN COVIDSeq Pipeline	Delta	B.1.617.2
LAZ-FPG-257	WGS	Illumina COVIDSeq assay	Illumina DRAGEN COVIDSeq Pipeline	Delta	B.1.617.2
LAZ-FPG-258	WGS	Illumina COVIDSeq assay	Illumina DRAGEN COVIDSeq Pipeline	Delta	B.1.617.2
LAZ-FPG-259	WGS	Illumina COVIDSeq assay	Illumina DRAGEN COVIDSeq Pipeline	Delta	B.1.617.2
LAZ-FPG-260	WGS	Illumina COVIDSeq assay	Illumina DRAGEN COVIDSeq Pipeline	Delta	B.1.617.2
LAZ-FPG-261	WGS	Illumina COVIDSeq assay	Illumina DRAGEN COVIDSeq Pipeline	Delta	B.1.617.2
LAZ-FPG-262	WGS	Illumina COVIDSeq assay	Illumina DRAGEN COVIDSeq Pipeline	Delta	B.1.617.2
LAZ-FPG-263	WGS	Illumina COVIDSeq assay	Illumina DRAGEN COVIDSeq Pipeline	Delta	B.1.617.2
LAZ-FPG-264	WGS	Illumina COVIDSeq assay	Illumina DRAGEN COVIDSeq Pipeline	Delta	B.1.617.2
LAZ-FPG-265	WGS	Illumina COVIDSeq assay	Illumina DRAGEN COVIDSeq Pipeline	Delta	B.1.617.2
LAZ-FPG-266	WGS	Illumina COVIDSeq assay	Illumina DRAGEN COVIDSeq Pipeline	Delta	B.1.617.2
LAZ-FPG-267	WGS	Illumina COVIDSeq assay	Illumina DRAGEN COVIDSeq Pipeline	Delta	B.1.617.2
LAZ-FPG-268	WGS	Illumina COVIDSeq assay	Illumina DRAGEN COVIDSeq Pipeline	Delta	B.1.617.2
LAZ-FPG-269	WGS	Illumina COVIDSeq assay	Illumina DRAGEN COVIDSeq Pipeline	Delta	B.1.617.2
LAZ-FPG-270	WGS	Illumina COVIDSeq assay	Illumina DRAGEN COVIDSeq Pipeline	Delta	B.1.617.2
LAZ-FPG-271	WGS	Illumina COVIDSeq assay	Illumina DRAGEN COVIDSeq Pipeline	Delta	B.1.617.2
LAZ-FPG-272	WGS	Illumina COVIDSeq assay	Illumina DRAGEN COVIDSeq Pipeline	Delta	B.1.617.2
LAZ-FPG-273	WGS	Illumina COVIDSeq assay	Illumina DRAGEN COVIDSeq Pipeline	Delta	B.1.617.2
LAZ-FPG-274	WGS	Illumina COVIDSeq assay	Illumina DRAGEN COVIDSeq Pipeline	Delta	B.1.617.2
LAZ-FPG-275	WGS	Illumina COVIDSeq assay	Illumina DRAGEN COVIDSeq Pipeline	Delta	B.1.617.2
LAZ-FPG-276	WGS	Illumina COVIDSeq assay	Illumina DRAGEN COVIDSeq Pipeline	Delta	B.1.617.2
LAZ-FPG-277	WGS	Illumina COVIDSeq assay	Illumina DRAGEN COVIDSeq Pipeline	Delta	B.1.617.2
LAZ-FPG-278	WGS	Illumina COVIDSeq assay	Illumina DRAGEN COVIDSeq Pipeline	Delta	B.1.617.2
LAZ-FPG-279	WGS	Illumina COVIDSeq assay	Illumina DRAGEN COVIDSeq Pipeline	Delta	B.1.617.2
LAZ-FPG-280	WGS	Illumina COVIDSeq assay	Illumina DRAGEN COVIDSeq Pipeline	Delta	B.1.617.2
LAZ-FPG-281	WGS	Illumina COVIDSeq assay	Illumina DRAGEN COVIDSeq Pipeline	Delta	B.1.617.2
LAZ-FPG-282	WGS	Illumina COVIDSeq assay	Illumina DRAGEN COVIDSeq Pipeline	Delta	B.1.617.2
LAZ-FPG-283	WGS	Illumina COVIDSeq assay	Illumina DRAGEN COVIDSeq Pipeline	Delta	B.1.617.2
LAZ-FPG-284	WGS	Illumina COVIDSeq assay	Illumina DRAGEN COVIDSeq Pipeline	Delta	B.1.617.2
LAZ-FPG-285	WGS	Illumina COVIDSeq assay	Illumina DRAGEN COVIDSeq Pipeline	Delta	B.1.617.2
LAZ-FPG-286	WGS	Illumina COVIDSeq assay	Illumina DRAGEN COVIDSeq Pipeline	Delta	B.1.617.2
LAZ-FPG-288	WGS	Illumina COVIDSeq assay	Illumina DRAGEN COVIDSeq Pipeline	Delta	B.1.617.2
LAZ-FPG-289	WGS	Illumina COVIDSeq assay	Illumina DRAGEN COVIDSeq Pipeline	Delta	B.1.617.2
LAZ-FPG-290	WGS	Illumina COVIDSeq assay	Illumina DRAGEN COVIDSeq Pipeline	Delta	B.1.617.2
LAZ-FPG-291	WGS	Illumina COVIDSeq assay	Illumina DRAGEN COVIDSeq Pipeline	Delta	B.1.617.2
LAZ-FPG-292	WGS	Illumina COVIDSeq assay	Illumina DRAGEN COVIDSeq Pipeline	Delta	B.1.617.2
LAZ-FPG-293	WGS	Illumina COVIDSeq assay	Illumina DRAGEN COVIDSeq Pipeline	Delta	B.1.617.2
LAZ-FPG-294	WGS	Illumina COVIDSeq assay	Illumina DRAGEN COVIDSeq Pipeline	Delta	B.1.617.2
LAZ-FPG-295	WGS	Illumina COVIDSeq assay	Illumina DRAGEN COVIDSeq Pipeline	Delta	B.1.617.2
LAZ-FPG-296	WGS	Illumina COVIDSeq assay	Illumina DRAGEN COVIDSeq Pipeline	Delta	B.1.617.2
LAZ-FPG-297	WGS	Illumina COVIDSeq assay	Illumina DRAGEN COVIDSeq Pipeline	Delta	B.1.617.2
LAZ-FPG-298	WGS	Illumina COVIDSeq assay	Illumina DRAGEN COVIDSeq Pipeline	Delta	B.1.617.2
LAZ-FPG-299	WGS	Illumina COVIDSeq assay	Illumina DRAGEN COVIDSeq Pipeline	Delta	B.1.617.2
LAZ-FPG-300	WGS	Illumina COVIDSeq assay	Illumina DRAGEN COVIDSeq Pipeline	Delta	B.1.617.2

LAZ-FPG-301	WGS	Illumina COVIDSeq assay	Illumina DRAGEN COVIDSeq Pipeline	Delta	B.1.617.2
LAZ-FPG-304	WGS	Illumina COVIDSeq assay	Illumina DRAGEN COVIDSeq Pipeline	Delta	B.1.617.2
LAZ-FPG-305	WGS	Illumina COVIDSeq assay	Illumina DRAGEN COVIDSeq Pipeline	Delta	B.1.617.2
LAZ-FPG-306	WGS	Illumina COVIDSeq assay	Illumina DRAGEN COVIDSeq Pipeline	Delta	B.1.617.2
LAZ-FPG-308	WGS	Illumina COVIDSeq assay	Illumina DRAGEN COVIDSeq Pipeline	Delta	B.1.617.2
LAZ-FPG-309	WGS	Illumina COVIDSeq assay	Illumina DRAGEN COVIDSeq Pipeline	Delta	B.1.617.2
LAZ-FPG-310	WGS	Illumina COVIDSeq assay	Illumina DRAGEN COVIDSeq Pipeline	Delta	B.1.617.2
LAZ-FPG-311	WGS	Illumina COVIDSeq assay	Illumina DRAGEN COVIDSeq Pipeline	Delta	B.1.617.2
LAZ-FPG-313	WGS	Illumina COVIDSeq assay	Illumina DRAGEN COVIDSeq Pipeline	Delta	B.1.617.2
LAZ-FPG-314	WGS	Illumina COVIDSeq assay	Illumina DRAGEN COVIDSeq Pipeline	Delta	B.1.617.2
LAZ-FPG-316	WGS	Illumina COVIDSeq assay	Illumina DRAGEN COVIDSeq Pipeline	Delta	B.1.617.2
LAZ-FPG-317	WGS	Illumina COVIDSeq assay	Illumina DRAGEN COVIDSeq Pipeline	Delta	B.1.617.2
LAZ-FPG-318	WGS	Illumina COVIDSeq assay	Illumina DRAGEN COVIDSeq Pipeline	Delta	B.1.617.2
LAZ-FPG-319	WGS	Illumina COVIDSeq assay	Illumina DRAGEN COVIDSeq Pipeline	Delta	B.1.617.2
LAZ-FPG-320	WGS	Illumina COVIDSeq assay	Illumina DRAGEN COVIDSeq Pipeline	Delta	B.1.617.2
LAZ-FPG-321	WGS	Illumina COVIDSeq assay	Illumina DRAGEN COVIDSeq Pipeline	Delta	B.1.617.2
LAZ-FPG-322	WGS	Illumina COVIDSeq assay	Illumina DRAGEN COVIDSeq Pipeline	Delta	B.1.617.2
LAZ-FPG-323	WGS	Illumina COVIDSeq assay	Illumina DRAGEN COVIDSeq Pipeline	Delta	B.1.617.2
LAZ-FPG-324	WGS	Illumina COVIDSeq assay	Illumina DRAGEN COVIDSeq Pipeline	Delta	B.1.617.2
LAZ-FPG-325	WGS	Illumina COVIDSeq assay	Illumina DRAGEN COVIDSeq Pipeline	Delta	B.1.617.2
LAZ-FPG-326	WGS	Illumina COVIDSeq assay	Illumina DRAGEN COVIDSeq Pipeline	Delta	B.1.617.2
LAZ-FPG-327	WGS	Illumina COVIDSeq assay	Illumina DRAGEN COVIDSeq Pipeline	Delta	B.1.617.2
LAZ-FPG-328	WGS	Illumina COVIDSeq assay	Illumina DRAGEN COVIDSeq Pipeline	Delta	B.1.617.2
LAZ-FPG-329	WGS	Illumina COVIDSeq assay	Illumina DRAGEN COVIDSeq Pipeline	Delta	B.1.617.2
LAZ-FPG-330	WGS	Illumina COVIDSeq assay	Illumina DRAGEN COVIDSeq Pipeline	Delta	B.1.617.2
LAZ-FPG-331	WGS	Illumina COVIDSeq assay	Illumina DRAGEN COVIDSeq Pipeline	Delta	B.1.617.2
LAZ-FPG-332	WGS	Illumina COVIDSeq assay	Illumina DRAGEN COVIDSeq Pipeline	Delta	B.1.617.2
LAZ-FPG-333	WGS	Illumina COVIDSeq assay	Illumina DRAGEN COVIDSeq Pipeline	Delta	B.1.617.2
LAZ-FPG-334	WGS	Illumina COVIDSeq assay	Illumina DRAGEN COVIDSeq Pipeline	Delta	B.1.617.2
LAZ-FPG-335	WGS	Illumina COVIDSeq assay	Illumina DRAGEN COVIDSeq Pipeline	Delta	B.1.617.2
LAZ-FPG-336	WGS	Illumina COVIDSeq assay	Illumina DRAGEN COVIDSeq Pipeline	Delta	B.1.617.2
LAZ-FPG-337	WGS	Illumina COVIDSeq assay	Illumina DRAGEN COVIDSeq Pipeline	Delta	B.1.617.2
LAZ-FPG-338	WGS	Illumina COVIDSeq assay	Illumina DRAGEN COVIDSeq Pipeline	Delta	B.1.617.2
LAZ-FPG-339	WGS	Illumina COVIDSeq assay	Illumina DRAGEN COVIDSeq Pipeline	Delta	B.1.617.2
LAZ-FPG-340	WGS	Illumina COVIDSeq assay	Illumina DRAGEN COVIDSeq Pipeline	Delta	B.1.617.2
LAZ-FPG-341	WGS	Illumina COVIDSeq assay	Illumina DRAGEN COVIDSeq Pipeline	Delta	B.1.617.2
LAZ-FPG-342	WGS	Illumina COVIDSeq assay	Illumina DRAGEN COVIDSeq Pipeline	Delta	B.1.617.2
LAZ-FPG-343	WGS	Illumina COVIDSeq assay	Illumina DRAGEN COVIDSeq Pipeline	Delta	B.1.617.2
LAZ-FPG-344	WGS	Illumina COVIDSeq assay	Illumina DRAGEN COVIDSeq Pipeline	Delta	B.1.617.2
LAZ-FPG-345	WGS	Illumina COVIDSeq assay	Illumina DRAGEN COVIDSeq Pipeline	Delta	B.1.617.2
LAZ-FPG-346	WGS	Illumina COVIDSeq assay	Illumina DRAGEN COVIDSeq Pipeline	Delta	B.1.617.2
LAZ-FPG-347	WGS	Illumina COVIDSeq assay	Illumina DRAGEN COVIDSeq Pipeline	Delta	B.1.617.2
LAZ-FPG-348	WGS	Illumina COVIDSeq assay	Illumina DRAGEN COVIDSeq Pipeline	Delta	B.1.617.2
LAZ-FPG-349	WGS	Illumina COVIDSeq assay	Illumina DRAGEN COVIDSeq Pipeline	Delta	B.1.617.2
LAZ-FPG-350	WGS	Illumina COVIDSeq assay	Illumina DRAGEN COVIDSeq Pipeline	Delta	B.1.617.2

LAZ-FPG-351	WGS	Illumina COVIDSeq assay	Illumina DRAGEN COVIDSeq Pipeline	Delta	B.1.617.2
LAZ-FPG-352	WGS	Illumina COVIDSeq assay	Illumina DRAGEN COVIDSeq Pipeline	Delta	B.1.617.2
LAZ-FPG-353	WGS	Illumina COVIDSeq assay	Illumina DRAGEN COVIDSeq Pipeline	Delta	B.1.617.2
LAZ-FPG-354	WGS	Illumina COVIDSeq assay	Illumina DRAGEN COVIDSeq Pipeline	Delta	B.1.617.2
LAZ-FPG-355	WGS	Illumina COVIDSeq assay	Illumina DRAGEN COVIDSeq Pipeline	Delta	B.1.617.2
LAZ-FPG-356	WGS	Illumina COVIDSeq assay	Illumina DRAGEN COVIDSeq Pipeline	Delta	B.1.617.2
LAZ-FPG-357	WGS	Illumina COVIDSeq assay	Illumina DRAGEN COVIDSeq Pipeline	Delta	B.1.617.2
LAZ-FPG-358	WGS	Illumina COVIDSeq assay	Illumina DRAGEN COVIDSeq Pipeline	Delta	B.1.617.2
LAZ-FPG-359	WGS	Illumina COVIDSeq assay	Illumina DRAGEN COVIDSeq Pipeline	Delta	B.1.617.2
LAZ-FPG-360	WGS	Illumina COVIDSeq assay	Illumina DRAGEN COVIDSeq Pipeline	Delta	B.1.617.2
LAZ-FPG-361	WGS	Illumina COVIDSeq assay	Illumina DRAGEN COVIDSeq Pipeline	Delta	B.1.617.2
LAZ-FPG-362	WGS	Illumina COVIDSeq assay	Illumina DRAGEN COVIDSeq Pipeline	Delta	B.1.617.2
LAZ-FPG-363	WGS	Illumina COVIDSeq assay	Illumina DRAGEN COVIDSeq Pipeline	Delta	B.1.617.2
LAZ-FPG-364	WGS	Illumina COVIDSeq assay	Illumina DRAGEN COVIDSeq Pipeline	Delta	B.1.617.2
LAZ-FPG-365	WGS	Illumina COVIDSeq assay	Illumina DRAGEN COVIDSeq Pipeline	Delta	B.1.617.2
LAZ-FPG-366	WGS	Illumina COVIDSeq assay	Illumina DRAGEN COVIDSeq Pipeline	Delta	B.1.617.2
LAZ-FPG-367	WGS	Illumina COVIDSeq assay	Illumina DRAGEN COVIDSeq Pipeline	Delta	B.1.617.2
LAZ-FPG-368	WGS	Illumina COVIDSeq assay	Illumina DRAGEN COVIDSeq Pipeline	Delta	B.1.617.2
LAZ-FPG-369	WGS	Illumina COVIDSeq assay	Illumina DRAGEN COVIDSeq Pipeline	Delta	B.1.617.2
LAZ-FPG-370	WGS	Illumina COVIDSeq assay	Illumina DRAGEN COVIDSeq Pipeline	Delta	B.1.617.2
LAZ-FPG-371	WGS	Illumina COVIDSeq assay	Illumina DRAGEN COVIDSeq Pipeline	Delta	B.1.617.2
LAZ-FPG-372	WGS	Illumina COVIDSeq assay	Illumina DRAGEN COVIDSeq Pipeline	Delta	B.1.617.2
LAZ-FPG-373	WGS	Illumina COVIDSeq assay	Illumina DRAGEN COVIDSeq Pipeline	Delta	B.1.617.2
LAZ-FPG-374	WGS	Illumina COVIDSeq assay	Illumina DRAGEN COVIDSeq Pipeline	Delta	B.1.617.2
LAZ-FPG-375	WGS	Illumina COVIDSeq assay	Illumina DRAGEN COVIDSeq Pipeline	Delta	B.1.617.2
LAZ-FPG-376	WGS	Illumina COVIDSeq assay	Illumina DRAGEN COVIDSeq Pipeline	Delta	B.1.617.2
LAZ-FPG-377	WGS	Illumina COVIDSeq assay	Illumina DRAGEN COVIDSeq Pipeline	Delta	B.1.617.2
LAZ-FPG-378	WGS	Illumina COVIDSeq assay	Illumina DRAGEN COVIDSeq Pipeline	Delta	B.1.617.2
LAZ-FPG-379	WGS	Illumina COVIDSeq assay	Illumina DRAGEN COVIDSeq Pipeline	Delta	B.1.617.2
LAZ-FPG-380	WGS	Illumina COVIDSeq assay	Illumina DRAGEN COVIDSeq Pipeline	Delta	B.1.617.2
LAZ-FPG-381	WGS	Illumina COVIDSeq assay	Illumina DRAGEN COVIDSeq Pipeline	Delta	B.1.617.2
LAZ-FPG-382	WGS	Illumina COVIDSeq assay	Illumina DRAGEN COVIDSeq Pipeline	Delta	B.1.617.2
LAZ-FPG-383	WGS	Illumina COVIDSeq assay	Illumina DRAGEN COVIDSeq Pipeline	Delta	B.1.617.2
LAZ-FPG-384	WGS	Illumina COVIDSeq assay	Illumina DRAGEN COVIDSeq Pipeline	Delta	B.1.617.2
LAZ-FPG-385	WGS	Illumina COVIDSeq assay	Illumina DRAGEN COVIDSeq Pipeline	Delta	B.1.617.2
LAZ-FPG-386	WGS	Illumina COVIDSeq assay	Illumina DRAGEN COVIDSeq Pipeline	Delta	B.1.617.2
LAZ-FPG-387	WGS	Illumina COVIDSeq assay	Illumina DRAGEN COVIDSeq Pipeline	Delta	B.1.617.2
LAZ-FPG-388	WGS	Illumina COVIDSeq assay	Illumina DRAGEN COVIDSeq Pipeline	Delta	B.1.617.2
LAZ-FPG-389	WGS	Illumina COVIDSeq assay	Illumina DRAGEN COVIDSeq Pipeline	Delta	B.1.617.2
LAZ-FPG-390	WGS	Illumina COVIDSeq assay	Illumina DRAGEN COVIDSeq Pipeline	Delta	B.1.617.2
LAZ-FPG-391	WGS	Illumina COVIDSeq assay	Illumina DRAGEN COVIDSeq Pipeline	Delta	B.1.617.2
LAZ-FPG-392	WGS	Illumina COVIDSeq assay	Illumina DRAGEN COVIDSeq Pipeline	Delta	B.1.617.2
LAZ-FPG-393	WGS	Illumina COVIDSeq assay	Illumina DRAGEN COVIDSeq Pipeline	Delta	B.1.617.2
LAZ-FPG-394	WGS	Illumina COVIDSeq assay	Illumina DRAGEN COVIDSeq Pipeline	Delta	B.1.617.2
LAZ-FPG-395	WGS	Illumina COVIDSeq assay	Illumina DRAGEN COVIDSeq Pipeline	Delta	B.1.617.2

LAZ-FPG-396	WGS	Illumina COVIDSeq assay	Illumina DRAGEN COVIDSeq Pipeline	Delta	B.1.617.2
LAZ-FPG-514	WGS	Illumina COVIDSeq assay	Illumina DRAGEN COVIDSeq Pipeline	Omicron	B.1.1.529.1
LAZ-FPG-515	WGS	Illumina COVIDSeq assay	Illumina DRAGEN COVIDSeq Pipeline	Omicron	B.1.1.529.1
LAZ-FPG-559	WGS	Illumina COVIDSeq assay	Illumina DRAGEN COVIDSeq Pipeline	Omicron	B.1.1.529.1
LAZ-FPG-560	WGS	Illumina COVIDSeq assay	Illumina DRAGEN COVIDSeq Pipeline	Omicron	B.1.1.529.1
LAZ-FPG-561	WGS	Illumina COVIDSeq assay	Illumina DRAGEN COVIDSeq Pipeline	Omicron	B.1.1.529.1
LAZ-FPG-562	WGS	Illumina COVIDSeq assay	Illumina DRAGEN COVIDSeq Pipeline	Omicron	B.1.1.529.1
LAZ-FPG-563	WGS	Illumina COVIDSeq assay	Illumina DRAGEN COVIDSeq Pipeline	Omicron	B.1.1.529.1
LAZ-FPG-564	WGS	Illumina COVIDSeq assay	Illumina DRAGEN COVIDSeq Pipeline	Omicron	B.1.1.529.1
LAZ-FPG-565	WGS	Illumina COVIDSeq assay	Illumina DRAGEN COVIDSeq Pipeline	Omicron	B.1.1.529.1
LAZ-FPG-566	WGS	Illumina COVIDSeq assay	Illumina DRAGEN COVIDSeq Pipeline	Omicron	B.1.1.529.1
LAZ-FPG-567	WGS	Illumina COVIDSeq assay	Illumina DRAGEN COVIDSeq Pipeline	Omicron	B.1.1.529.1
LAZ-FPG-568	WGS	Illumina COVIDSeq assay	Illumina DRAGEN COVIDSeq Pipeline	Omicron	B.1.1.529.1
LAZ-FPG-569	WGS	Illumina COVIDSeq assay	Illumina DRAGEN COVIDSeq Pipeline	Omicron	B.1.1.529.1
LAZ-FPG-571	WGS	Illumina COVIDSeq assay	Illumina DRAGEN COVIDSeq Pipeline	Omicron	B.1.1.529.1
LAZ-FPG-572	WGS	Illumina COVIDSeq assay	Illumina DRAGEN COVIDSeq Pipeline	Omicron	B.1.1.529.1
LAZ-FPG-573	WGS	Illumina COVIDSeq assay	Illumina DRAGEN COVIDSeq Pipeline	Omicron	B.1.1.529.1
LAZ-FPG-574	WGS	Illumina COVIDSeq assay	Illumina DRAGEN COVIDSeq Pipeline	Omicron	B.1.1.529.1
LAZ-FPG-576	WGS	Illumina COVIDSeq assay	Illumina DRAGEN COVIDSeq Pipeline	Omicron	B.1.1.529.1
LAZ-FPG-577	WGS	Illumina COVIDSeq assay	Illumina DRAGEN COVIDSeq Pipeline	Omicron	B.1.1.529.1
LAZ-FPG-578	WGS	Illumina COVIDSeq assay	Illumina DRAGEN COVIDSeq Pipeline	Omicron	B.1.1.529.1
LAZ-FPG-580	WGS	Illumina COVIDSeq assay	Illumina DRAGEN COVIDSeq Pipeline	Omicron	B.1.1.529.1
LAZ-FPG-582	WGS	Illumina COVIDSeq assay	Illumina DRAGEN COVIDSeq Pipeline	Omicron	B.1.1.529.1
LAZ-FPG-583	WGS	Illumina COVIDSeq assay	Illumina DRAGEN COVIDSeq Pipeline	Omicron	B.1.1.529.1
LAZ-FPG-586	WGS	Illumina COVIDSeq assay	Illumina DRAGEN COVIDSeq Pipeline	Omicron	B.1.1.529.1
LAZ-FPG-587	WGS	Illumina COVIDSeq assay	Illumina DRAGEN COVIDSeq Pipeline	Omicron	B.1.1.529.1
LAZ-FPG-588	WGS	Illumina COVIDSeq assay	Illumina DRAGEN COVIDSeq Pipeline	Omicron	B.1.1.529.1
LAZ-FPG-589	WGS	Illumina COVIDSeq assay	Illumina DRAGEN COVIDSeq Pipeline	Omicron	B.1.1.529.1
LAZ-FPG-590	WGS	Illumina COVIDSeq assay	Illumina DRAGEN COVIDSeq Pipeline	Omicron	B.1.1.529.1
LAZ-FPG-591	WGS	Illumina COVIDSeq assay	Illumina DRAGEN COVIDSeq Pipeline	Omicron	B.1.1.529.1
LAZ-FPG-593	WGS	Illumina COVIDSeq assay	Illumina DRAGEN COVIDSeq Pipeline	Omicron	B.1.1.529.1
LAZ-FPG-594	WGS	Illumina COVIDSeq assay	Illumina DRAGEN COVIDSeq Pipeline	Omicron	B.1.1.529.1
LAZ-FPG-595	WGS	Illumina COVIDSeq assay	Illumina DRAGEN COVIDSeq Pipeline	Omicron	B.1.1.529.1
LAZ-FPG-596	WGS	Illumina COVIDSeq assay	Illumina DRAGEN COVIDSeq Pipeline	Omicron	B.1.1.529.1
LAZ-FPG-598	WGS	Illumina COVIDSeq assay	Illumina DRAGEN COVIDSeq Pipeline	Omicron	B.1.1.529.1
LAZ-FPG-601	WGS	Illumina COVIDSeq assay	Illumina DRAGEN COVIDSeq Pipeline	Omicron	B.1.1.529.1
LAZ-FPG-602	WGS	Illumina COVIDSeq assay	Illumina DRAGEN COVIDSeq Pipeline	Omicron	B.1.1.529.1
LAZ-FPG-603	WGS	Illumina COVIDSeq assay	Illumina DRAGEN COVIDSeq Pipeline	Omicron	B.1.1.529.1
LAZ-FPG-604	WGS	Illumina COVIDSeq assay	Illumina DRAGEN COVIDSeq Pipeline	Omicron	B.1.1.529.1
LAZ-FPG-606	WGS	Illumina COVIDSeq assay	Illumina DRAGEN COVIDSeq Pipeline	Omicron	B.1.1.529.1
LAZ-FPG-607	WGS	Illumina COVIDSeq assay	Illumina DRAGEN COVIDSeq Pipeline	Omicron	B.1.1.529.1
LAZ-FPG-608	WGS	Illumina COVIDSeq assay	Illumina DRAGEN COVIDSeq Pipeline	Omicron	B.1.1.529.1
LAZ-FPG-609	WGS	Illumina COVIDSeq assay	Illumina DRAGEN COVIDSeq Pipeline	Omicron	B.1.1.529.1
LAZ-FPG-610	WGS	Illumina COVIDSeq assay	Illumina DRAGEN COVIDSeq Pipeline	Omicron	B.1.1.529.1
LAZ-FPG-612	WGS	Illumina COVIDSeq assay	Illumina DRAGEN COVIDSeq Pipeline	Omicron	B.1.1.529.1

LAZ-FPG-613	WGS	Illumina COVIDSeq assay	Illumina DRAGEN COVIDSeq Pipeline	Omicron	B.1.1.529.1
LAZ-FPG-614	WGS	Illumina COVIDSeq assay	Illumina DRAGEN COVIDSeq Pipeline	Omicron	B.1.1.529.1
LAZ-FPG-615	WGS	Illumina COVIDSeq assay	Illumina DRAGEN COVIDSeq Pipeline	Omicron	B.1.1.529.1
LAZ-FPG-617	WGS	Illumina COVIDSeq assay	Illumina DRAGEN COVIDSeq Pipeline	Omicron	B.1.1.529.1
LAZ-FPG-618	WGS	Illumina COVIDSeq assay	Illumina DRAGEN COVIDSeq Pipeline	Omicron	B.1.1.529.1
LAZ-FPG-619	WGS	Illumina COVIDSeq assay	Illumina DRAGEN COVIDSeq Pipeline	Omicron	B.1.1.529.1
LAZ-FPG-691	WGS	Illumina COVIDSeq assay	Illumina DRAGEN COVIDSeq Pipeline	Omicron	B.1.1.529.1
LAZ-FPG-692	WGS	Illumina COVIDSeq assay	Illumina DRAGEN COVIDSeq Pipeline	Omicron	B.1.1.529.1
LAZ-FPG-693	WGS	Illumina COVIDSeq assay	Illumina DRAGEN COVIDSeq Pipeline	Omicron	B.1.1.529.1
LAZ-FPG-695	WGS	Illumina COVIDSeq assay	Illumina DRAGEN COVIDSeq Pipeline	Omicron	B.1.1.529.1
LAZ-FPG-696	WGS	Illumina COVIDSeq assay	Illumina DRAGEN COVIDSeq Pipeline	Omicron	B.1.1.529.1
LAZ-FPG-697	WGS	Illumina COVIDSeq assay	Illumina DRAGEN COVIDSeq Pipeline	Omicron	B.1.1.529.1
LAZ-FPG-701	WGS	Illumina COVIDSeq assay	Illumina DRAGEN COVIDSeq Pipeline	Omicron	B.1.1.529.1
LAZ-FPG-702	WGS	Illumina COVIDSeq assay	Illumina DRAGEN COVIDSeq Pipeline	Omicron	B.1.1.529.1
LAZ-FPG-703	WGS	Illumina COVIDSeq assay	Illumina DRAGEN COVIDSeq Pipeline	Omicron	B.1.1.529.1
LAZ-FPG-706	WGS	Illumina COVIDSeq assay	Illumina DRAGEN COVIDSeq Pipeline	Omicron	B.1.1.529.1
LAZ-FPG-707	WGS	Illumina COVIDSeq assay	Illumina DRAGEN COVIDSeq Pipeline	Omicron	B.1.1.529.1
LAZ-FPG-708	WGS	Illumina COVIDSeq assay	Illumina DRAGEN COVIDSeq Pipeline	Omicron	B.1.1.529.1
LAZ-FPG-709	WGS	Illumina COVIDSeq assay	Illumina DRAGEN COVIDSeq Pipeline	Omicron	B.1.1.529.1
LAZ-FPG-710	WGS	Illumina COVIDSeq assay	Illumina DRAGEN COVIDSeq Pipeline	Omicron	B.1.1.529.1
LAZ-FPG-711	WGS	Illumina COVIDSeq assay	Illumina DRAGEN COVIDSeq Pipeline	Omicron	B.1.1.529.1
LAZ-FPG-712	WGS	Illumina COVIDSeq assay	Illumina DRAGEN COVIDSeq Pipeline	Omicron	B.1.1.529.1
LAZ-FPG-714	WGS	Illumina COVIDSeq assay	Illumina DRAGEN COVIDSeq Pipeline	Omicron	B.1.1.529.1
LAZ-FPG-715	WGS	Illumina COVIDSeq assay	Illumina DRAGEN COVIDSeq Pipeline	Omicron	B.1.1.529.1
LAZ-FPG-716	WGS	Illumina COVIDSeq assay	Illumina DRAGEN COVIDSeq Pipeline	Omicron	B.1.1.529.1
LAZ-FPG-717	WGS	Illumina COVIDSeq assay	Illumina DRAGEN COVIDSeq Pipeline	Omicron	B.1.1.529.1
LAZ-FPG-718	WGS	Illumina COVIDSeq assay	Illumina DRAGEN COVIDSeq Pipeline	Omicron	B.1.1.529.1
LAZ-FPG-719	WGS	Illumina COVIDSeq assay	Illumina DRAGEN COVIDSeq Pipeline	Omicron	B.1.1.529.1
LAZ-FPG-720	WGS	Illumina COVIDSeq assay	Illumina DRAGEN COVIDSeq Pipeline	Omicron	B.1.1.529.1
LAZ-FPG-721	WGS	Illumina COVIDSeq assay	Illumina DRAGEN COVIDSeq Pipeline	Omicron	B.1.1.529.1
LAZ-FPG-722	WGS	Illumina COVIDSeq assay	Illumina DRAGEN COVIDSeq Pipeline	Omicron	B.1.1.529.1
LAZ-FPG-723	WGS	Illumina COVIDSeq assay	Illumina DRAGEN COVIDSeq Pipeline	Omicron	B.1.1.529.1
LAZ-FPG-724	WGS	Illumina COVIDSeq assay	Illumina DRAGEN COVIDSeq Pipeline	Omicron	B.1.1.529.1
V1	Spike-NGS	Arrow Diagnostics SARS-CoV-2 S-gene	SmartSeq Covid Analyst	Alpha	
V10	Spike-NGS	Arrow Diagnostics SARS-CoV-2 S-gene	SmartSeq Covid Analyst	Alpha	
V100	Spike-NGS	Arrow Diagnostics SARS-CoV-2 S-gene	SmartSeq Covid Analyst	Alpha	
V101	Spike-NGS	Arrow Diagnostics SARS-CoV-2 S-gene	SmartSeq Covid Analyst	Alpha	
V110	Spike-NGS	Arrow Diagnostics SARS-CoV-2 S-gene	SmartSeq Covid Analyst	Gamma	
V12	Spike-NGS	Arrow Diagnostics SARS-CoV-2 S-gene	SmartSeq Covid Analyst	Alpha	
V137	Spike-NGS	Arrow Diagnostics SARS-CoV-2 S-gene	SmartSeq Covid Analyst	Alpha	
V14	Spike-NGS	Arrow Diagnostics SARS-CoV-2 S-gene	SmartSeq Covid Analyst	Alpha	
V140	Spike-NGS	Arrow Diagnostics SARS-CoV-2 S-gene	SmartSeq Covid Analyst	Gamma	
V155	Spike-NGS	Arrow Diagnostics SARS-CoV-2 S-gene	SmartSeq Covid Analyst	Alpha	
V162	Spike-NGS	Arrow Diagnostics SARS-CoV-2 S-gene	SmartSeq Covid Analyst	Alpha	
V163	Spike-NGS	Arrow Diagnostics SARS-CoV-2 S-gene	SmartSeq Covid Analyst	Alpha	

V164	Spike-NGS	Arrow Diagnostics SARS-CoV-2 S-gene	SmartSeq Covid Analyst	Gamma
V17	Spike-NGS	Arrow Diagnostics SARS-CoV-2 S-gene	SmartSeq Covid Analyst	Alpha
V18	Spike-NGS	Arrow Diagnostics SARS-CoV-2 S-gene	SmartSeq Covid Analyst	Gamma
V184	Spike-NGS	Arrow Diagnostics SARS-CoV-2 S-gene	SmartSeq Covid Analyst	Alpha
V189	Spike-NGS	Arrow Diagnostics SARS-CoV-2 S-gene	SmartSeq Covid Analyst	Gamma
V19	Spike-NGS	Arrow Diagnostics SARS-CoV-2 S-gene	SmartSeq Covid Analyst	Alpha
V193	Spike-NGS	Arrow Diagnostics SARS-CoV-2 S-gene	SmartSeq Covid Analyst	Gamma
V198	Spike-NGS	Arrow Diagnostics SARS-CoV-2 S-gene	SmartSeq Covid Analyst	Gamma
V20	Spike-NGS	Arrow Diagnostics SARS-CoV-2 S-gene	SmartSeq Covid Analyst	Alpha
V21	Spike-NGS	Arrow Diagnostics SARS-CoV-2 S-gene	SmartSeq Covid Analyst	Alpha
V215	Spike-NGS	Arrow Diagnostics SARS-CoV-2 S-gene	SmartSeq Covid Analyst	Gamma
V22	Spike-NGS	Arrow Diagnostics SARS-CoV-2 S-gene	SmartSeq Covid Analyst	Beta
V229	Spike-NGS	Arrow Diagnostics SARS-CoV-2 S-gene	SmartSeq Covid Analyst	Gamma
V23	Spike-NGS	Arrow Diagnostics SARS-CoV-2 S-gene	SmartSeq Covid Analyst	Alpha
V232	Spike-NGS	Arrow Diagnostics SARS-CoV-2 S-gene	SmartSeq Covid Analyst	Gamma
V238	Spike-NGS	Arrow Diagnostics SARS-CoV-2 S-gene	SmartSeq Covid Analyst	Gamma
V24	Spike-NGS	Arrow Diagnostics SARS-CoV-2 S-gene	SmartSeq Covid Analyst	Alpha
V241	Spike-NGS	Arrow Diagnostics SARS-CoV-2 S-gene	SmartSeq Covid Analyst	Gamma
V243	Spike-NGS	Arrow Diagnostics SARS-CoV-2 S-gene	SmartSeq Covid Analyst	Alpha
V244	Spike-NGS	Arrow Diagnostics SARS-CoV-2 S-gene	SmartSeq Covid Analyst	Alpha
V247	Spike-NGS	Arrow Diagnostics SARS-CoV-2 S-gene	SmartSeq Covid Analyst	Gamma
V259	Spike-NGS	Arrow Diagnostics SARS-CoV-2 S-gene	SmartSeq Covid Analyst	Gamma
V272	Spike-NGS	Arrow Diagnostics SARS-CoV-2 S-gene	SmartSeq Covid Analyst	Gamma
V275	Spike-NGS	Arrow Diagnostics SARS-CoV-2 S-gene	SmartSeq Covid Analyst	Gamma
V279	Spike-NGS	Arrow Diagnostics SARS-CoV-2 S-gene	SmartSeq Covid Analyst	Gamma
V288	Spike-NGS	Arrow Diagnostics SARS-CoV-2 S-gene	SmartSeq Covid Analyst	Beta
V29	Spike-NGS	Arrow Diagnostics SARS-CoV-2 S-gene	SmartSeq Covid Analyst	Alpha
V30	Spike-NGS	Arrow Diagnostics SARS-CoV-2 S-gene	SmartSeq Covid Analyst	Alpha
V309	Spike-NGS	Arrow Diagnostics SARS-CoV-2 S-gene	SmartSeq Covid Analyst	Gamma
V310	Spike-NGS	Arrow Diagnostics SARS-CoV-2 S-gene	SmartSeq Covid Analyst	Gamma
V312	Spike-NGS	Arrow Diagnostics SARS-CoV-2 S-gene	SmartSeq Covid Analyst	Delta
V315	Spike-NGS	Arrow Diagnostics SARS-CoV-2 S-gene	SmartSeq Covid Analyst	Alpha
V317	Spike-NGS	Arrow Diagnostics SARS-CoV-2 S-gene	SmartSeq Covid Analyst	Gamma
V318	Spike-NGS	Arrow Diagnostics SARS-CoV-2 S-gene	SmartSeq Covid Analyst	Gamma
V32	Spike-NGS	Arrow Diagnostics SARS-CoV-2 S-gene	SmartSeq Covid Analyst	Gamma
V320	Spike-NGS	Arrow Diagnostics SARS-CoV-2 S-gene	SmartSeq Covid Analyst	Delta
V323	Spike-NGS	Arrow Diagnostics SARS-CoV-2 S-gene	SmartSeq Covid Analyst	Delta
V352	Spike-NGS	Arrow Diagnostics SARS-CoV-2 S-gene	SmartSeq Covid Analyst	Delta
V353	Spike-NGS	Arrow Diagnostics SARS-CoV-2 S-gene	SmartSeq Covid Analyst	Delta
V355	Spike-NGS	Arrow Diagnostics SARS-CoV-2 S-gene	SmartSeq Covid Analyst	Delta
V361	Spike-NGS	Arrow Diagnostics SARS-CoV-2 S-gene	SmartSeq Covid Analyst	Delta
V362	Spike-NGS	Arrow Diagnostics SARS-CoV-2 S-gene	SmartSeq Covid Analyst	Delta
V364	Spike-NGS	Arrow Diagnostics SARS-CoV-2 S-gene	SmartSeq Covid Analyst	Delta
V365	Spike-NGS	Arrow Diagnostics SARS-CoV-2 S-gene	SmartSeq Covid Analyst	Delta
V366	Spike-NGS	Arrow Diagnostics SARS-CoV-2 S-gene	SmartSeq Covid Analyst	Delta

V367	Spike-NGS	Arrow Diagnostics SARS-CoV-2 S-gene	SmartSeq Covid Analyst	Delta
V39	Spike-NGS	Arrow Diagnostics SARS-CoV-2 S-gene	SmartSeq Covid Analyst	Gamma
V5	Spike-NGS	Arrow Diagnostics SARS-CoV-2 S-gene	SmartSeq Covid Analyst	Alpha
V6	Spike-NGS	Arrow Diagnostics SARS-CoV-2 S-gene	SmartSeq Covid Analyst	Alpha
V79	Spike-NGS	Arrow Diagnostics SARS-CoV-2 S-gene	SmartSeq Covid Analyst	Gamma
V81	Spike-NGS	Arrow Diagnostics SARS-CoV-2 S-gene	SmartSeq Covid Analyst	Gamma
V96	Spike-NGS	Arrow Diagnostics SARS-CoV-2 S-gene	SmartSeq Covid Analyst	Gamma

TABLE S2 Seegene assay parameters' percentages per the SARS-CoV-2 variant detection in NPS samples as calculated in comparison with the NGS-based reference method[a]

Type of variant (no. of samples with the identified variant by the NGS method)	Sensitivity	Specificity	Positive predictive value	Negative predictive value
	**Seegene Variants I assay**			
Alpha (37)	100 (37/37)	100 (251/251)	100 (37/37)	100 (251/251)
Beta (2)	NC (0/2)	99.6 (286/287)	NC (0/1)	99.3 (286/288)
Gamma (28)	100 (28/28)	99.6 (260/261)	96.5 (28/29)	100 (260/260)
Delta (221)	99.6 (220/221)	100 (67/67)	100 (220/220)	98.5 (67/68)
	**Seegene Variants I and II assays**			
Omicron (77)	79.2 (61/77)	NA	100 (61/61)	NA

[a]To calculate the listed parameters for each variant detection, we considered as true negative all the samples that were not identified as positive for the variant of interest. For example, regarding the Alpha variant, we calculated the number of true-negative samples as the sum of all positive samples for Beta, Gamma, or Delta variants (n = 251). Additionally, we considered one Delta variant-positive sample, which was misidentified as a Beta/Gamma variant-positive sample, as false negative for calculating the Delta detection sensitivity or as false positive for calculating both the Beta/Gamma detection specificity and positive predictive value. For the Omicron variant-positive samples, we could not calculate specificity and negative predictive value because of the lack of true-negative samples. NA, not applicable; NC, not calculable; NGS, next generation sequencing; NPS, NPS, nasopharyngeal swab; SARS-CoV-2, severe acute respiratory syndrome coronavirus 2.
